# SurfFold: a unified model for protein inverse folding by integrating surface and structural information

**DOI:** 10.1093/bioinformatics/btaf666

**Published:** 2025-12-19

**Authors:** Darong Li, Lian Shen, Meijia Song, Deyi Li, Juan Liu, Xiangrong Liu

**Affiliations:** Department of Computer Science and Technology, Xiamen University, Xiamen, 361005, China; Department of Computer Science and Technology, Xiamen University, Xiamen, 361005, China; Department of Computer Science and Technology, Xiamen University, Xiamen, 361005, China; Department of Computer Science and Technology, Xiamen University, Xiamen, 361005, China; Pen-Tung Sah Institute of Micro-Nano Science and Technology, Xiamen University, Xiamen, 361005, China; Department of Computer Science and Technology, Xiamen University, Xiamen, 361005, China; State Key Laboratory of Vaccines for Infectious Diseases, Xiang An Biomedicine Laboratory, Xiamen, 361005, China

## Abstract

**Motivation:**

Proteins play a crucial role in biological systems, and accurate protein sequence prediction is essential for applications such as drug discovery. Existing inverse folding models primarily rely on protein backbone structure information, overlooking the biochemical properties embedded in protein surface data that constrain its functionality, leading to limited prediction accuracy.

**Results:**

We propose a novel inverse folding framework, SurfFold, which integrates both protein backbone structure and surface information for sequence prediction. Additionally, it incorporates side-chain structural information and its interaction with surface information. Then, we introduce a Representation alignment module to better fuze structure and surface Representations. Experimental results demonstrate that SurfFold achieves state-of-the-art performance on the CATH4.2 dataset, and additional experiments validate the effectiveness of the proposed modules. Moreover, the homologous structure inverse folding experiment also demonstrates that SurfFold possesses excellent capability in homologous protein design.

**Availability and implementation:**

The source code and data are available at https://github.com/jiudizhengf/SurfFold.

## 1 Introduction

Proteins play an indispensable role in biological systems, including providing structural support, catalyzing metabolic reactions, mediating molecular transport, transmitting cellular signals, and supporting immune defense. Therefore, precise protein design is crucial for advancing applications in drug discovery, biocatalysis, materials science, agricultural biotechnology, and environmental remediation (Huang *et al.* 2016). Protein design generally comprises two core aspects: structure design and sequence design. In recent years, considerable progress has been achieved in predicting protein structures from amino acid sequences (Senior *et al.* 2020, Jumper *et al.* 2021). Models such as AlphaFold (Jumper *et al.* 2021) and ESMFold (Lin *et al.* 2023) have significantly improved the accuracy and efficiency of protein tertiary structure prediction. Despite these advances, the inverse problem of predicting amino acid sequences from known protein structures, known as inverse folding, remains a persistent challenge.

Traditional inverse folding methods often rely on energy functions to directly model the protein folding state. While these approaches reflect the underlying physical principles, they are computationally expensive and heavily dependent on expert prior knowledge (Onuchic *et al.* 1997, Dill and MacCallum 2012). In recent years, deep learning techniques have been widely applied to inverse folding tasks, significantly improving predictive performance (Gao *et al.* 2023b, 2024b, Yang *et al.* 2023). For example, Ingraham *et al.* (2019) developed the GraphTrans model, which enables bidirectional prediction of protein structures and sequences using a graph attention network. Gao *et al.* (2022) proposed AlphaDesign, which integrates graph neural networks with the AlphaFold (Jumper *et al.* 2021) structure prediction tool to generate sequences given a target structure. Zheng *et al.* (2023) introduced LM-design, incorporating structural adapters into protein language models to effectively integrate 3D Structure information. Gao *et al.* (2023a) also designed PiFold, which combines innovative feature extractors with a graph neural network architecture to enable one-shot generation of protein sequences. In addition, models such as ProteinMPNN (Dauparas *et al.* 2022), GVP (Jing *et al.* 2020), GCA (Tan *et al.* 2022), ESM-IF (Hsu *et al.* 2022), and ScFold (Zhong *et al.* 2025) have shown strong performance in inverse folding tasks. However, these methods focus primarily on protein structure information while neglecting biochemical information. In homologous protein design, using only structure templates overlooks how protein surface biochemistry affects function. This leads to suboptimal results (Bertoni *et al.* 2017). Crucially, biochemical information on protein surfaces plays a vital role in protein functionality.

In the other field of protein representation learning, researchers have successfully enhanced prediction tasks by employing multi-modal protein representations that integrate multi-modal information of proteins. For instance, Yuan *et al.* (2024) introduced the ProteinF3S model, which combines sequence, structure, and surface information to improve the accuracy of enzyme function prediction. Xu *et al.* (2024) developed the MFE framework, utilizing multi-modal feature extraction to enhance the precision of protein–ligand affinity prediction. Additional methods, including DeepProSite (Fang *et al.* 2023), EquiPNAS (Roche *et al.* 2024), and GeoBind (Li and Liu 2023), further demonstrate the effectiveness of integrating multiple modalities for protein feature modeling.

This multi-modal paradigm is particularly critical for the inverse folding task, where the objective is to design protein sequences that fold into specific 3D structures and fulfill intended functional roles. Achieving functional accuracy necessitates capturing not only the geometric constraints imposed by the backbone structure but also integrating the biochemical constraints encoded on the protein surface, which govern molecular interactions and functionality (Song *et al.* 2024). Although protein surface representations inherently contain rich biochemical information (Song *et al.* 2024) and are derived from the underlying 3D structure, naively combining discrete structure representations with continuous surface representations often results in spatial reference frame misalignment and semantic inconsistencies between geometric and biochemical representations. This spatial and semantic misalignment hinders the model’s ability to learn a unified representation that faithfully reflects the intricate coupling between structure and surface biochemistry. The SurfPro model (Song *et al.* 2024), e.g., demonstrated the value of surface biochemical attributes for sequence prediction but did not explicitly integrate geometric structure representations, leaving the challenge of reconciling these two critical modalities unresolved.

To effectively bridge this gap and construct a cohesive multi-modal representation for inverse folding, we leverage side-chain conformations. Side-chains reside precisely at the interface between structure and surface: their geometric conformations are constrained by the backbone scaffold, while their chemical identities and spatial arrangements directly define the physicochemical properties of the protein surface (Zhu *et al.* 2012). Moreover, these conformations are profoundly influenced and shaped by the local biochemical environment (Rose 2019). Consequently, utilizing side-chain information provides a natural and biophysically grounded mechanism to integrate geometric structural constraints with surface biochemical information, thereby establishing a more expressive and functionally predictive framework for protein sequence design.

Motivated by these considerations, we propose SurfFold, a novel inverse folding framework that integrates protein structural and surface representations. We first adapt the three-level feature extraction strategy introduced in ProNet (Wang *et al.* 2022) to obtain backbone and side-chain structural information. We then generate the protein surface using the dMaSIF (Sverrisson *et al.* 2021) framework. Our architecture includes three main components: a feature encoding module, a representation alignment module, and a one-shot decoding module. The backbone and side-chain information are jointly encoded using E(n)-equivariant graph neural networks (EGNNs) (Satorras *et al.* 2021), while surface features are processed using geodesic convolution. Spatial correspondence between surface points and Alpha Carbon ($C_{\alpha }$) atoms is then established based on Euclidean distances to achieve precise feature alignment. Finally, a one-shot decoder is used to generate the predicted amino acid sequence. Experimental results demonstrate that SurfFold achieves state-of-the-art performance on the CATH4.2 dataset (Orengo *et al.* 1997). The model consistently achieves sequence-recovery rates of 62.47% in the test dataset. Additional experiments validate the critical role of the representation alignment module, and ablation studies confirm the necessity and effectiveness of each component within the framework. Furthermore, the homologous structure inverse folding experiment establishes SurfFold’s excellent performance in designing homologous proteins.

## 2 Materials and methods

**Figure 1.**
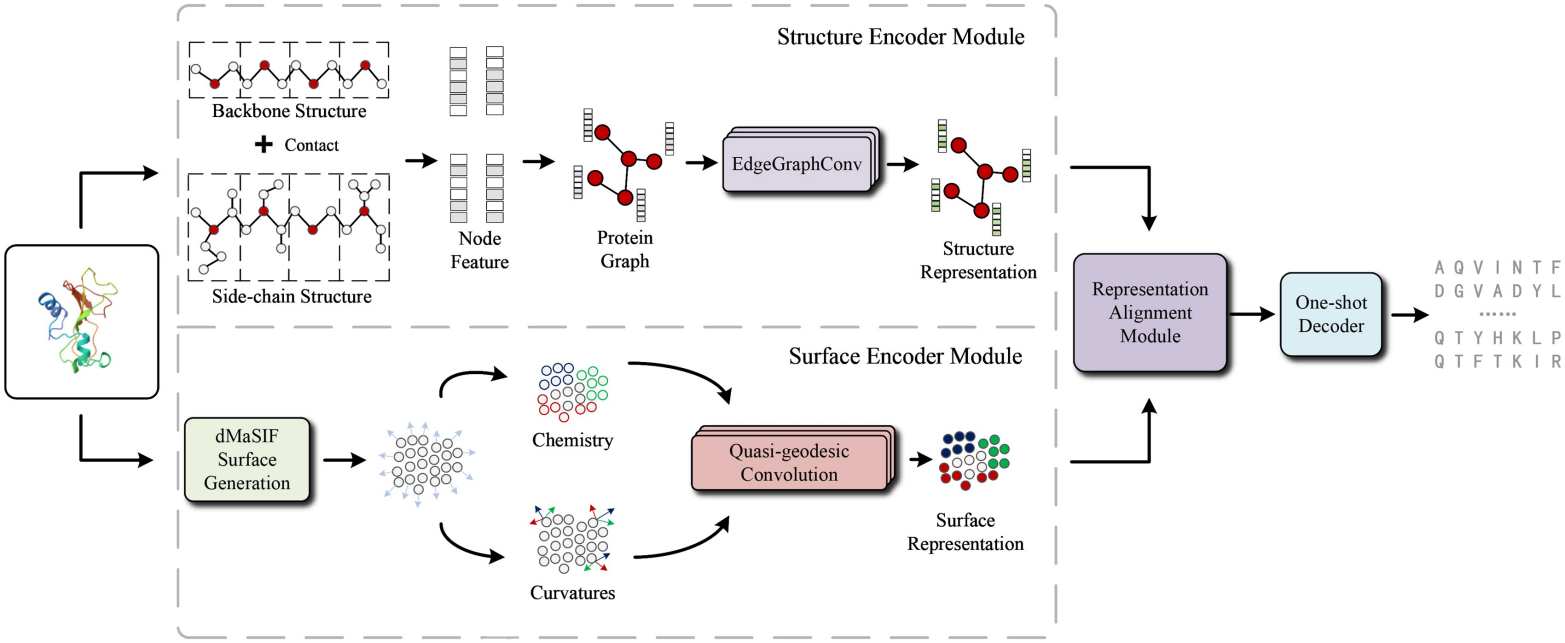
The overall framework of SurfFold. The Structure Encoder Module extracts representations from the protein structure. The Surface Encoder Module utilizes the dMaSIF surface generation method to generate the protein surface from its structure and then extracts surface representations. The Representation Fusion Module integrates representations from these two modalities to form a fused representation. This fused representation is ultimately decoded via one-shot decoding to predict the protein sequence.

In this section, we provide detailed implementation specifics of our proposed SurfFold. SurfFold integrates protein surface information with structural information to predict protein sequences. Additionally, we propose a representation alignment method to unify the protein surface representations and structural representations obtained from distinct modules. Thus, the implementation of SurfFold primarily consists of three modules: Structure Encoder Module, Surface Encoder Module, Representation Alignment Module. Figure 1 depicts the SurfFold architecture, highlighting the interactions among the three modules.

### 2.1 Structure Encoder Module

In contrast to most inverse folding models, our Structure Encoder Module leverages both backbone and side-chain structures of proteins. Using atomic coordinates from PDB files, we extract the backbone and side-chain structure to construct the initial node features of the protein graph. Within the Structure Encoder Module, we construct a protein graph based on distances between $C_{\alpha }$ atoms, establishing an edge between two $C_{\alpha }$ nodes if their distance falls below a certain threshold. Subsequently, the combined backbone and side-chain features serve as initial node representations, which are processed by an EdgeGraphConv (Wang *et al.* 2022) network to generate residue-level structural representations. EdgeGraphConv is similar to GraphConv (Morris *et al.* 2019), with the only difference being that EdgeGraphConv applies the Hadamard product between node features and edge features.

#### 2.1.1 Backbone structure features

Following the backbone structure feature extraction approach described in ProNet (Wang *et al.* 2022), we define a local coordinate system for each amino acid residue. Each amino acid residue is represented as a node in the graph, and we extract spherical coordinates ($d_{ji},\theta_{ji},\phi_{ji}$) of node *j* in the local coordinate system of node *i*, as well as the rotation angle $\omega_{ji}$ of the edge between these nodes, to serve as basic backbone structural features. Furthermore, to determine the relative orientation between two amino acids, we calculate three Euler angles ($\tau_{ji}^{1},\tau_{ji}^{2},\tau_{ji}^{3}$) between their respective backbone coordinate systems. These Euler angles represent the relative orientation between the two amino acid backbone coordinate systems and help to describe their spatial relationship. The angles and coordinate information are then combined to form the structural features of the protein backbone


(1) $$\mathrm{Featur}e_{b}=(d_{ji},\theta_{ji},\phi_{ji})\cup (\tau_{ji}^{1},\tau_{ji}^{2},\tau_{ji}^{3})$$

where $\mathrm{Featur}e_{b}$ is the feature of protein backbone structure.

#### 2.1.2 Side-chain structure features

To effectively integrate with protein surface information, we adopt the side-chain structural feature extraction method described in ProNet (Wang *et al.* 2022) during our data preprocessing stage. We specifically extract structural information from the protein side chains without incorporating detailed atomic information, thus avoiding potential data leakage issues. Based on known bond lengths and bond angles among atoms within side chains, we calculate four side-chain torsion angles ($\chi_{1},\chi_{2},\chi_{3},\chi_{4}$). These torsion angles are subsequently integrated with backbone structural information to generate the initial node features for the protein graph


(2) $$\mathrm{Featur}e_{p}=\mathrm{Featur}e_{b}\cup (\chi_{1},\chi_{2},\chi_{3},\chi_{4})$$

where $\mathrm{Featur}e_{p}$ is the node feature of protein graph.

### 2.2 Surface Encoder Module

To address critical information that protein internal structures fail to capture, SurfFold introduces a Surface Encoder Module to thoroughly exploit information from the protein surface. The Surface Encoder Module leverages the dMaSIF (Sverrisson *et al.* 2021) method, significantly enhancing both the efficiency and accuracy of surface generation compared to traditional methods such as MSMS (Ewing and Hermisson 2010) and MaSIF (Gainza *et al.* 2020). Initially, the module performs protein surface sampling, subsequently extracting chemical (such as electrostatic properties) and geometric attributes (such as surface curvature) from the generated surface point cloud. Finally, quasi-geodesic convolutions integrate and effectively learn the surface information, resulting in robust protein surface representations.

#### 2.2.1 Surface generation

Protein surface reconstruction is directly based on atomic coordinates and types, avoiding explicit mesh preprocessing. Utilizing the Smooth Distance Function (SDF) (Sverrisson *et al.* 2021), we define the protein surface as an isosurface. Initial points are generated via Gaussian sampling and projected onto the isosurface by gradient optimization. The resulting point cloud is then downsampled to yield a uniformly distributed protein surface point cloud. The SDF is defined as:


(3) $$\sigma (x)=\frac{\sum_{k=1}^{A}\;\text{exp}\;\left(-\frac{∥x-a_{k}∥}{\sigma_{k}}\right)\sigma_{k}}{\sum_{k=1}^{A}\;\text{exp}\;\left(-\frac{∥x-a_{k}∥}{\sigma_{k}}\right)}$$

(4) $$\text{SDF}(x)=-\sigma (x)\cdot \;\text{log}\;\left(\sum_{k=1}^{A}\;\text{exp}\;\left(-\frac{∥x-a_{k}∥}{\sigma_{k}}\right)\right)$$

where $\sigma_{k}$ denotes the radius of atom $a_{k}$. Subsequently, the normals for each point on the surface are computed by normalizing the gradient of the SDF:


(5) $${\hat{n}}_{i}=\frac{\nabla \text{SDF}(x_{i})}{∥\nabla \text{SDF}(x_{i})∥}$$

where the ∇ denotes the Gradient operator. This point cloud-based generation method reduces preprocessing time and significantly improves computational efficiency for large-scale protein data.

#### 2.2.2 Surface chemical features

During chemical feature extraction, our approach eschews traditional handcrafted descriptors, such as electrostatic potentials, instead employing deep networks to autonomously learn chemical environmental information. For each surface point, we identify its 16 nearest atoms. The atomic types (encoded via one-hot encoding) and the inverse distances to the surface points are used as input features. These features are first processed by a small MLP, and then passed through another MLP to yield the final chemical features. This end-to-end, data-driven strategy enables adaptive learning of diverse protein surface environments and effectively characterizes essential chemical properties, demonstrating superior performance in dMaSIF (Sverrisson *et al.* 2021) benchmarks.

#### 2.2.3 Surface structure features

For structural feature extraction, the module emphasizes capturing local geometric information. Considering each surface point as a reference, we compute multi-scale curvature features (mean curvature and Gaussian curvature) from neighboring points. These curvatures effectively describe both detailed and overall surface shape at various scales. The structural features obtained are concatenated with corresponding chemical features to serve as inputs to subsequent convolutional layers. By adjusting Gaussian weight windows and quasi-geodesic neighborhood sizes, the model adeptly adapts to different local geometric patterns and maintains robustness against spatial rotations and translations, thereby improving the model’s ability to capture and characterize complex protein surface structures.

#### 2.2.4 Quasi-geodesic convolution

After obtaining chemical and geometric surface features, quasi-geodesic convolutions capture local spatial structural features and map neighboring point features into a higher-dimensional space. The convolution operation selects neighboring points using Gaussian weighting, approximating geodesic distances by considering spatial distances and angles between normals. Neighboring point features are encoded within local coordinate systems and aggregated via a trainable MLP to form new point features. This approach is invariant to 3D spatial rotations and translations, while effectively exploiting complex surface spatial information.

### 2.3 Representation alignment module

**Figure 2.**
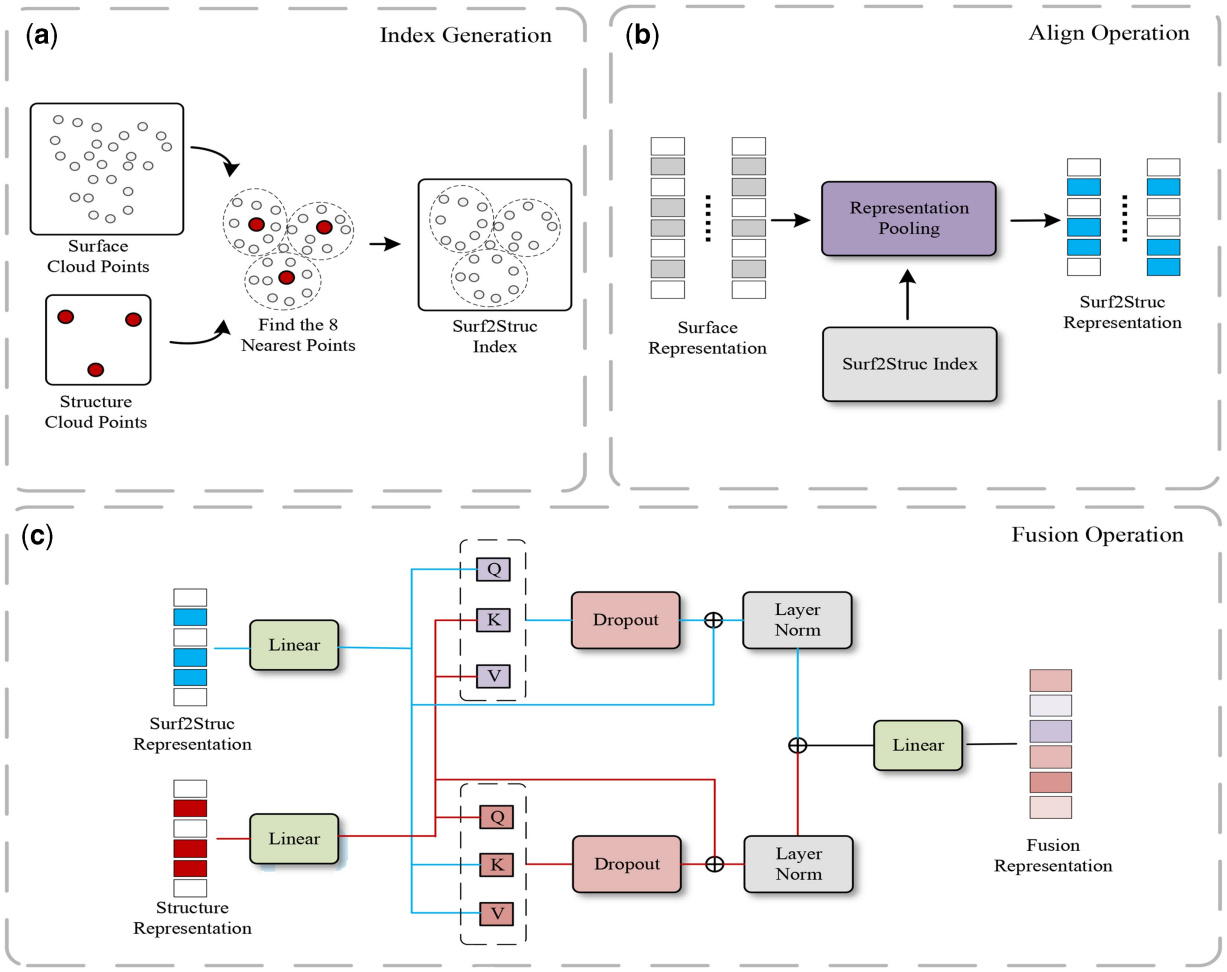
(a) The 8-nearest-neighbor alignment operation. During the data processing stage, the model first identifies the eight nearest surface point clouds for each structural point cloud based on their 3D coordinates. These indices are stored as Surf2Struc index. (b and c) Constitute the representation alignment module, where (b) represents the surface representation alignment operation. Using the precomputed Surf2Struc indices, this module processes the surface representation (from the Surface Encoder Module) to align them with the structure representation (from the Structure Encoder Module), thereby generating Surf2Struc representation with identical dimensions. (c) Refers to the representation fusion operation within the Representation Alignment Module. The model employs bidirectional cross-attention to fuze the structure representations and Surf2Struc representation into a unified Fusion representation, which is subsequently used for one-shot decoding to predict the protein sequence.

The number of protein surface points generated by dMaSIF (Sverrisson *et al.* 2021) is substantially larger than the number of structural points in the protein backbone. After learning separate representations from the surface and structural modules, fuzing these representations poses challenges in both geometric alignment and semantic consistency. To address this, we design a representation fusion module that integrates outputs from the two branches to facilitate downstream protein sequence prediction. The architecture of this module is illustrated in Fig. 2.

First, we use a KDTree to identify the eight nearest surface points for each $C_{\alpha }$ atom in the protein backbone, ensuring efficient nearest-neighbor search. The representations of these eight surface points are aggregated through average pooling to generate a surface representation aligned with each corresponding $C_{\alpha }$ atom. This operation ensures that the number of surface representations matches that of the structural nodes. Subsequently, we employ separate MLPs to project the structural and surface representations into a shared dimension space:


(6) $$\{\begin{array}{c} a^{\prime }=W_{a}a+b_{a},\\ b^{\prime }=W_{b}b+b_{b} \end{array}$$

where a and b denote the structural and surface representations, respectively, and $W_{a}$, $W_{b}$, $b_{a}$, and $b_{b}$ are learnable parameters.

To further integrate the protein structure and surface information, we apply a cross-attention mechanism to jointly refine both representations:


(7) $$\{\begin{array}{c} {\text{Attn}}_{A}=\text{MHA}(a^{\prime },b^{\prime },b^{\prime }),\\ {\text{Attn}}_{B}=\text{MHA}(b^{\prime },a^{\prime },a^{\prime }) \end{array}$$

where MHA(·) denotes the multi-head attention operation, with the first argument as the query, and the second and third arguments as the key and value, respectively.

We then apply residual connections and layer normalization to the attention outputs, resulting in enhanced fused representations. Finally, the fused representation used for sequence prediction is generated by passing through an MLP:


(8) $$\begin{array}{c} a_{1}=\text{LayerNorm}\left(a^{\prime }+\text{Dropout}({\text{Attn}}_{A})\right),\\ b_{1}=\text{LayerNorm}\left(b^{\prime }+\text{Dropout}({\text{Attn}}_{B})\right),\\ f_{\text{fused}}={\text{MLP}}_{\text{fusion}}\left(\left[a_{1};b_{1}\right]\right) \end{array}$$

where [·;·] denotes concatenation along the feature dimension.

### 2.4 One-shot decoder

To generate the protein sequence from the fused representation, we adapt a one-shot decoder inspired by PiFold (Gao *et al.* 2023a). Unlike autoregressive decoders, which predict amino acids sequentially and often require beam search, the one-shot decoder predicts the entire sequence simultaneously, significantly improving computational efficiency. Despite its simplicity, PiFold (Gao *et al.* 2023a) demonstrates that this approach achieves competitive sequence-recovery accuracy. Following this design, we implement our decoder as a multi-layer perceptron (MLP) that maps the fused representation $f_{\text{fused}}$ directly to a probability distribution over the amino acid vocabulary at each position. In addition, this approach reduces the risk of exposure bias commonly associated with autoregressive decoding, as each amino acid prediction is conditionally independent given the fused representation. This approach strikes a balance between decoding speed and prediction quality, making it ideal for large-scale inverse folding applications.

## 3 Results and Discussion

### 3.1 Baseline

To ensure a fair and comprehensive evaluation, we compare our proposed inverse folding model with several representative state-of-the-art methods. The selected baselines include both classical algorithms and advanced deep learning models. These consist of graph neural network-based methods such as GVP (Jing *et al.* 2020), GCA (Tan *et al.* 2022), and StructGNN (Tan *et al.* 2022), as well as recently developed neural frameworks like ProteinMPNN (Dauparas *et al.* 2022), LM-design (Zheng *et al.* 2023), PiFold (Gao *et al.* 2023a), Knowledge-Design (Gao *et al.* 2024a), and ScFold (Zhong *et al.* 2025). Additionally, we include SurfPro (Song *et al.* 2024) as a surface-based method and ESM-IF (Hsu *et al.* 2022) as a powerful pretrained model. Together, these baselines represent diverse algorithmic paradigms in the field of protein sequence design, facilitating a comprehensive and objective evaluation of our model’s performance and generalization capability. To ensure a fair comparison with existing methods, we adopt the same evaluation metrics and dataset as PiFold (Gao *et al.* 2023a). The experimental data are the average of results across different random seeds. Further dataset descriptions and evaluation metrics are provided in the Supplementary Materials, available as supplementary data at *Bioinformatics* online.

### 3.2 Result

The experimental results on CATH4.2 (Orengo *et al.* 1997), shown in Table 1, demonstrate that SurfFold achieves the lowest perplexity across all test partitions, benefiting from the integration of 3D structural and protein surface information. In terms of sequence recovery, SurfFold attains 56.18%, 54.38%, and 62.05% on short-chain, single-chain, and full-test partitions, respectively. While the gain over the second-best model is modest (1.3%) on the full-test set, SurfFold shows notable advantages on short-chain and single-chain partitions, suggesting that its effectiveness is largely independent of protein sequence length. Moreover, compared with SurfPro (Song *et al.* 2024), which uses only surface information, SurfFold improves sequence recovery by 4.27%, highlighting the value of combining structural and surface features for more accurate protein sequence prediction.

**Table 1.** CATH4.2 dataset experiments.

Model	Perplexity ↓			Recovery ↑		
	Short	Single	All	Short	Single	All
GVP	7.23	7.84	5.36	30.60	28.95	39.47
GCA	7.09	7.49	6.05	32.62	31.10	37.64
StructGNN	8.29	8.74	6.40	29.44	28.26	35.91
ProteinMPNN	6.21	6.68	4.61	36.35	34.43	45.96
LM-design	6.77	6.46	4.52	37.88	42.47	55.65
ESM-IF	8.18	6.33	6.44	31.3	38.5	38.3
PiFold	6.04	6.31	4.55	39.84	38.53	51.66
SurfPro	–	–	3.13	–	–	57.78
Knowledge-Design	5.48	5.1	3.46	44.66	45.45	60.77
ScFold	5.80	5.99	4.61	41.60	40.10	52.22
SurfFold(our)	**3.20** ± **0.06**	**3.32** ± **0.03**	**2.95** ± **0.01**	**56.18** ± **1.27**	**54.38** ± **0.34**	**62.05** ± **0.54**

**Note:** The best performance is highlighted in bold.

In Table 2, SurfFold achieves the lowest perplexity (2.90/2.74) and the highest sequence recovery (64.16%/69.32%). Compared with the prior best, Knowledge-Design, SurfFold improves recovery by 1.37 percentage points on TS50 and still leads by 0.13 points on TS500. Against strong baselines on TS50, it gains +9.73 and +5.44 points over ProteinMPNN and PiFold, respectively, indicating that fuzing 3D structural and surface cues markedly enhances sequence design quality. Taken together, these consistent gains across small (TS50) and medium (TS500) benchmarks suggest strong generalization across dataset scales and protein diversity.

**Table 2.** TS50 and TS500 dataset experiments.

Model	TS50		TS500	
	Perlexity	Recovery	Perlexity	Recovery
GVP	4.71	44.14	4.20	49.14
GCA	5.09	47.02	4.72	47.74
StructGNN	5.40	43.89	4.98	45.69
ProteinMPNN	3.93	54.43	3.53	58.08
PiFold	3.86	58.72	3.44	60.42
Knowledge-Design	3.10	62.79	2.86	69.19
ScFold	3.71	59.32	–	61.59
SurfFold(our)	**2.90** ± **0.05**	**64.16** ± **0.27**	**2.74** ± **0.02**	**69.32** ± **0.40**

**Note:** The best performance is highlighted in bold.

### 3.3 Ablation studies

We conducted a series of ablation studies to rigorously assess the contribution of each component in our model and understand their individual impacts on performance. Based on the architecture described in Section 2, we constructed several variant models. Regarding the protein modalities, we evaluated the following variants in addition to the full model: (1) a backbone-only variant, (2) a backbone with surface variant, and (3) a backbone with side-chain variant. At the module level, we also tested versions that omit the 8-nearest-neighbor aggregation for surface representation and those that exclude the bidirectional cross-attention mechanism for integrating surface and structural features. The performance of each ablated model is summarized in Table 3.

**Table 3.** Ablation experiments.

		SurfFold	Model1	Model2	Model3	Model4	Model5
Protein features	Backbone	√	√	√	√	√	√
	Side-chain	√	√			√	√
	Surface	√			√	√	√
Alignment Module	8-nearest alignment	√			√	√	
	Cross-attention	√			√		√
Results	Perplexity	2.95±0.01	3.23±0.02	13.27±0.36	8.20±0.06	3.24±0.02	3.12±0.03
	Recovery	62.05±0.54	56.94±0.62	18.31±1.52	36.03±0.96	57.61±0.53	60.04±1.21

The ablation results reveal that side-chain information alone yields a substantial performance gain, yet it is insufficient to achieve state-of-the-art accuracy. Only when side-chain features are effectively fused with surface representations does the model realize its full potential. Moreover, these experiments demonstrate that our proposed 8-nearest-neighbor aggregation and bidirectional cross-attention fusion significantly mitigate conflicts between different modal embeddings, thereby enhancing overall model performance.

### 3.4 Representation alignment analysis and visualization

**Figure 3.**
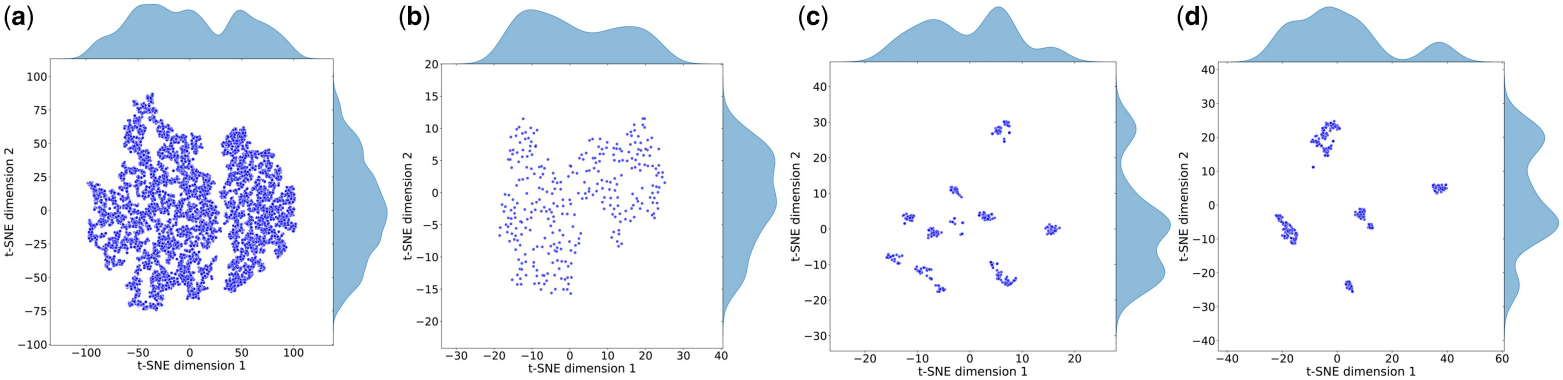
(a) The initial protein surface data. (b) The Surf2Struc representation after the 8-nearest-neighbor alignment. (c) The protein structural data. (d) The fusion representation after the representation alignment module.

To further assess the efficacy of our representation alignment module, we visualized representations immediately before and after its application. Using saved model parameters, we randomly sampled test instances and projected them into two dimensions using t-SNE (Fig. 3). In these representations, the raw surface representation appears as a diffuse, unstructured cloud, reflecting the fact that the surface point cloud vastly outnumbers the structural point cloud and that surface descriptors are much higher in dimension, both of which introduce considerable noise. After applying aggregation of the eight nearest neighbors, the surface representation shows better local coherence and more closely mirrors the structural representation, with irrelevant noise effectively reduced. Nonetheless, although both types of representations tend to form clusters, significant class overlap remains. In contrast, once structure and surface representation are fused using cross-attention, the fused representation reveals clusters that are clearly separated. These results confirm that our alignment module substantially improves the discriminative power of the feature space, boosting inter-class separability and reducing noise, which in turn enables the one-shot decoder to predict protein sequences with greater accuracy.

### 3.5 Homologous structure inverse folding experiment analysis

To better validate the role of the protein surface and side chains introduced by SurfFold, we designed the homologous structure inverse folding experiment. Homologous structure inverse folding refers to the computational inverse design of amino acid sequences capable of folding into spatial conformations closely resembling a given homologous structural template, thereby enabling directed optimization or *de novo* functionalization of specific biological properties. We conducted pairwise structural TM-score analysis for all proteins in the test set, selecting pairs with a TM-score between 0.3 and 0.6. From these, we randomly sampled 1000 pairs based on TM-score distribution and grouped them into bins with a 0.02 interval. We then predicted the sequence of one protein in each pair using SurfFold and PiFold, comparing it to the native sequence of the other protein to measure sequence similarity. We also calculated the native sequence similarity between the protein pairs and computed the average relative difference between predicted and native similarity for each group. The average relative difference measures the relative error between the predicted sequence and the true sequence, defined as the difference in percent identity between the two, divided by the percent identity of the true sequence. The detail is shown in Supplementary Materials, available as supplementary data at *Bioinformatics* online.

**Figure 4.**
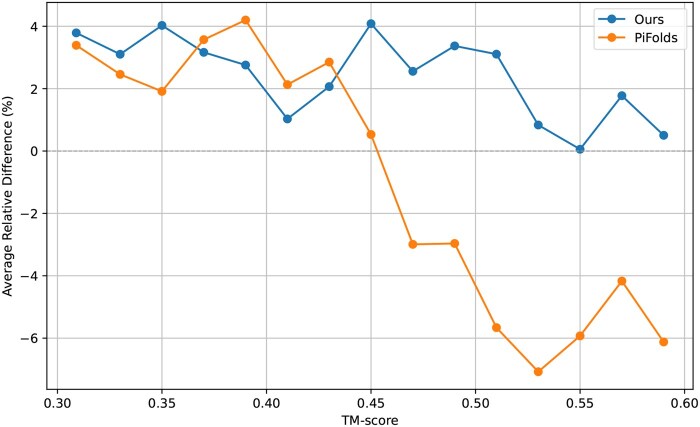
Relative difference between predicted and true protein sequence by TM-score.

Figure 4 compares how the sequence prediction accuracy of the two protein inverse folding models [SurfFold and PiFold (Gao *et al.* 2023a)] varies with TM-score (structural similarity). The performance of SurfFold and PiFold (Gao *et al.* 2023a) is comparable when the TM-score is between 0.3 and 0.45. However, when the TM-score exceeds 0.45, the sequence similarity predicted by SurfFold becomes significantly higher than that predicted by PiFold (Gao *et al.* 2023a). Backbone-only geometry is insufficient for highly similar protein structures because it ignores surface chemical cues, causing key-residue mismatches and lowering accuracy. By jointly modeling surface geometry and chemistry (electrostatics, hydrophobicity), SurfFold captures constraints at functional sites—e.g., assigning Arg/Lys to negatively charged patches—and improves prediction. Even in medium-homology cases, conserved local physicochemical microenvironments guide SurfFold to place property-matched residues (e.g. Phe/Tyr in hydrophobic pockets), strengthening homology-based prediction and design.

## 4 Conclusion

This article presents a novel protein sequence prediction framework, SurfFold, which enhances sequence prediction accuracy by integrating protein structural information and surface information. SurfFold combines backbone, side-chain structures, and protein surface data, utilizing E(3)-equivariant neural networks and geodesic convolution networks to extract structural and surface features, respectively. We introduce a representation-alignment module to reconcile structural and surface embeddings, which markedly improves predictive performance. On CATH4.2 (Orengo *et al.* 1997), SurfFold surpasses prior models, with especially strong sequence-recovery gains on short- and single-chain proteins; alignment visualizations and homologous inverse folding results further confirm the benefit of jointly modeling structural and surface cues. Currently limited to proteins, the framework has not yet been tested on nucleic acids or protein–nucleic acid complexes; future work will extend SurfFold to these settings.

## Supplementary Material

btaf666_Supplementary_Data

## Data Availability

The source code and data are available at https://github.com/jiudizhengf/SurfFold
