# Supplementary material for: SurfFold: a unified model for protein inverse folding by integrating surface and structural information
Source: Bioinformatics. 2025 Dec 19;42(1):btaf666. doi: 10.1093/bioinformatics/btaf666 (PMC12857571; doi:10.1093/bioinformatics/btaf666)
Supplement: btaf666_Supplementary_Data [file btaf666_supplementary_data.pdf]

Supplementary Data For Paper: SurfFold: A Unified Model for Protein Inverse Folding by Integrating Surface and Structural Information

1 Surface Generation

1.1 Fast Sampling

The protein surface is modeled using an atomic point cloud. To represent this surface accurately, we employ a smooth distance function, often referred to as a metaball. For each atom, 20 points are sampled in a Gaussian distribution around its position. The smooth distance function $\mathrm{SDF}(x)$ defines the interaction between the points and the atomic centers:

$$\mathrm{SDF}(x) = -\sigma(x) \cdot \log\left(\sum_{k=1}^{A} \exp\left(-\frac{\|x - a_k\|}{\sigma_k}\right)\right)$$

Here, $\sigma(x)$ represents the average atomic radius in the neighborhood of point x, computed as:

$$\sigma(x) = \frac{\sum_{k=1}^{A} \exp\left(-\frac{\|x-a_k\|}{\sigma_k}\right)\sigma_k}{\sum_{k=1}^{A} \exp\left(-\frac{\|x-a_k\|}{\sigma_k}\right)}$$

After generating the points, we apply gradient descent to adjust their positions and minimize the difference from the target radius $r = 1.05$ Å by using a squared loss function. The points are then filtered based on distance criteria, retaining those within the target range $r \pm 0.1$ Å, ensuring that their positions are refined after multiple gradient descent steps.

1.2 Construction of the Local Coordinate System

For each sampled point x_i, the gradient of the smooth distance function is normalized to obtain the unit normal vector $\hat{n}_i$. To establish a local coordinate system $(\hat{n}_i, \hat{u}_i, \hat{v}_i)$, we smooth the normal vectors using a Gaussian kernel with smoothing parameter $\sigma = 9$ Å:

$$\hat{n}_i = \mathrm{Normalize}\left(\sum_{j=1}^{N} \exp\left(-\frac{\|x_i - x_j\|^2}{2\sigma^2}\right)\hat{n}_j\right)$$

The tangent vectors $\hat{u}_i$ and $\hat{v}_i$ are then calculated as follows, where $\hat{n}_i = [x, y, z]$:

$$\hat{u}_i = \left[1 + sax^2, sb, -sx\right], \quad \hat{v}_i = \left[b, s + ay^2, -y\right]$$

In these expressions, $s = \mathrm{sign}(z)$, $a = -\frac{1}{s+z}$, and $b = axy$, defining the local coordinate system for each point on the protein surface.

1.3 Chemical Features

To capture the chemical characteristics of the surface, we first identify the 16 nearest atomic centers $\{a_1^i, ..., a_{16}^i\}$ for each point x_i, and encode their atomic types $\{t_1^i, ..., t_{16}^i\}$ as one-hot vectors in $\mathbb{R}^4$.

These feature vectors are passed through a multi-layer perceptron (MLP) to generate local chemical feature vectors $C_{i,k} \in \mathbb{R}^{16}$. The sum of these vectors from the nearest neighbors is computed, and a second MLP is applied to produce the final chemical feature vector $C_i \in \mathbb{R}^{16}$, capturing the local chemical environment of each sampled surface point.

2 Evaluation Metrics

2.1 Perplexity and Recovery

To comprehensively assess the performance of our inverse folding model, we employ two widely used evaluation metrics: perplexity and recovery. Perplexity measures the uncertainty in the predicted amino acid distribution and reflects the model's ability to generate plausible protein sequences; a lower perplexity indicates that the predicted sequences are more coherent and better aligned with the statistical properties of natural protein sequences. Given the ground-truth sequence $y = (y_1, y_2, ..., y_N)$ and the predicted sequence $\hat{y} = (\hat{y_1}, \hat{y_2}, ..., \hat{y_N})$, perplexity is defined as:

$$\text{Perplexity} = \exp\left(-\frac{1}{N}\sum_{i=1}^{N} \log P(y_i \mid \hat{y_i})\right) \tag{1}$$

where N denotes the sequence length and $P(y_i \mid \hat{y_i})$ is the model's predicted probability for residue y_i at position i conditioned on the previous residues. Recovery evaluates the proportion of residues in the predicted sequence that exactly match those in the native structure, and is defined as:

$$\text{Recovery} = \frac{1}{N}\sum_{i=1}^{N} \mathbb{I}(y_i = \hat{y_i}) \tag{2}$$

where $\mathbb{I}$ is the indicator function. A higher recovery score indicates that the model is more capable of accurately reconstructing the original sequence, thereby demonstrating its practical potential for protein sequence design. Together, these two metrics provide a comprehensive and rigorous evaluation of sequence quality and accuracy in the inverse folding task.

2.2 Average Relative Difference

Given two aligned sequences aln_seq1 and aln_seq2, where each pair of characters at position i in the sequences is denoted as a_i and b_i (for $1 \leq i \leq N$, with N being the length of the alignment):

$$\text{Percent Identity(aln\_seq1, aln\_seq2)} = \frac{\sum_{i=1}^{N} \mathbf{1}(a_i = b_i \text{ and } a_i \neq' -' \text{ and } b_i \neq' -')}{N} \times 100$$

Where $\mathbf{1}(\cdot)$ is the indicator function, which equals 1 when the condition is true, and 0 otherwise. Given a pair of protein sequences, where one protein is used as the reference sequence (seq1), and the other protein sequence is the target sequence (seq2), with the sequence predicted by the network being seq3.

$$\text{ID1} = \text{Percent Identity}(seq1, seq2)$$

$$\text{ID2} = \text{Percent Identity}(seq1, seq3)$$

$$\text{average relative difference} = \frac{ID1 - ID2}{ID1}$$

3 Dataset

3.1 CATH4.2

CATH4.2 is a large and widely used protein structure dataset that covers a vast number of 3D protein structures and is organized in detail based on the CATH classification system. The CATH system employs a four-level classification hierarchy: Class, Architecture, Topology, and Homologous Superfamily. Each level is carefully annotated, ranging from broad structural categories to detailed homologous superfamilies, making this dataset of great value in areas such as protein structure prediction, functional analysis, and homologous modeling. CATH4.2 provides researchers with a large amount of structural data that supports various types of protein analyses, making it particularly suitable for cross-species and functional protein structure comparison studies.

**Dataset**	**Number of Samples**
Training Set	18024
Validation Set	608
Test Set	1120

Table 1: Experimental Partitioning of CATH4.2 Dataset

3.2 TS50 and TS500

The TS50 dataset is a smaller dataset containing 50 protein structures, specifically designed for TS50 is a small dataset containing 50 protein structures with meticulous annotations covering representative folds and functional features, making it suitable for early-stage training, debugging, and small-sample evaluation of algorithms; TS500 contains 500 three-dimensional protein structures, with a larger sample size, more complex structural and functional information, and a richer variety of fold types (e.g., α-helices, β-sheets, and their combinations), making it better suited for training more complex models and method optimization. Together, the two datasets support different stages of protein structure research—from preliminary experiments to large-scale modeling—and complement each other.

**Dataset**	**Number of Samples**
Training Set	18024
TS50 Test Set	50
TS500 Test Set	500

Table 2: Experimental Partitioning of TS50 and TS500 Dataset

4 Module Input and Output

4.1 Structure Encoder Module

The Structure Encoder Module focuses on encoding the structural information of the input protein structure. The details are shown in Table 3.

Table 3: Structure Encoder Module Input and Output Dimension

Module	Input	Output
Structure Extraction	atoms_xyz: 3	sidechain_embed: 8<br>backbone_embed: 6
Generate Protein Graph	atoms_xyz: 3<br>sidechain_embed: 8<br>backbone_embed: 6	sidechain_feature: 36<br>backbone_feature: 24
EdgeGraphConv	sidechain_feature: 36<br>backbone_feature: 24	Structure Representation: 128

4.2 Surface Encoder Module

The Surface Encoder Module processes the surface-related properties of the structure. The details are shown in Table 4.

Table 4: Surface Encoder Module Input and Output Dimension

Module	Input	Output
Surface Generation	atoms_xyz: 3	surface_xyz: 3<br>normals: 3<br>struct2surf_index: 8
Generate Chemistry and Curvatures	surface_xyz: 3<br>normals: 3<br>atoms_xyz: 3	chemistry_feature: 16<br>Curvature_feature: 10
Quasi-geodesic Convolution	chemistry_feature: 16<br>Curvature_feature: 10	Surface Repreasentation: 128

4.3 Alignment and Decoder Module

The Alignment and Decoder Module performs the fusion and decoding of the surface and structure representations. The details are shown in Table 5.

Table 5: Alignment and Decoder Module Input and Output Dimension

Module	Input	Output
Representation Alignment Module	Surface Repreasentation: 128<br>Structure Repreasentation: 128	Fusion Representation: 128
One-shot Decoder	Fusion Representation: 128	Final Output: 20

5 Supplementary Experiment

5.1 Efficiency Experiment

We provide supplemental information on training cost and inference latency. All measurements were performed on a single NVIDIA GeForce RTX 3090 GPU.

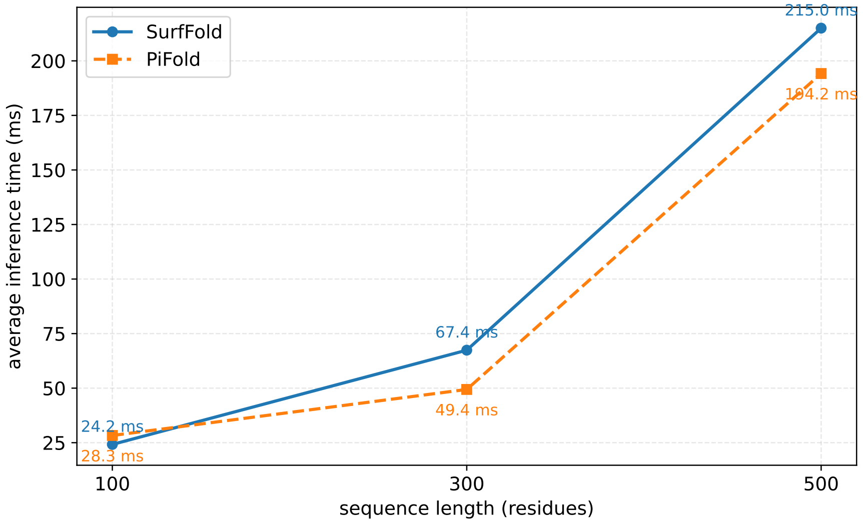

Figure 1: SurfFold vs PiFold — Comparison of Average Inference Time at Different Sequence Lengths

Training cost: SurfFold training wall-clock time was 2880 seconds (0.80 GPU-hours); PiFold training wall-clock time was 386 seconds (0.11 GPU-hours).

Inference latency (wall-clock time, average per protein, batch size = 1). Timings were measured after several warm-up runs and record only the model forward pass, minimizing I/O and data-loading overhead:

Sequence length 100 residues: SurfFold 24.2 ms/protein, PiFold 28.3 ms/protein.
Sequence length 300 residues: SurfFold 67.4 ms/protein, PiFold 49.4 ms/protein.
Sequence length 500 residues: SurfFold 215.0 ms/protein, PiFold 194.2 ms/protein.

As shown in Figure 1, SurfFold has a higher training cost than PiFold (0.80 vs 0.11 GPU-hours). For inference, SurfFold is slightly faster on short sequences (100 residues), while for medium and long sequences (300 and 500 residues) the two models have comparable latencies, with PiFold being somewhat faster at 300 and 500 residues.

5.2 Ablation Experiment for k

Regarding the question of why we chose k=8, we actually conducted ablation studies when selecting hyperparameters. During the ablation experiments, due to the high computational cost of k=16, we reduced the batch size to 4 for these specific tests. It is important to note that the experiments reported in the main text were conducted with a batch size of 16. Therefore, the results of the ablation study differ from those in the main text and subsequent analyses under batch size 16.

Table 6: Ablation Experiment for k

k	Surface Generation	Training	GPU Memory	Recovery
4	0.1317s	2787s	2.46GB	58.43%
8	0.1465s	2969s	3.87GB	58.97%
16	0.4879s	3301s	6.82GB	54.64%

Based on these results, k=16 not only requires substantially greater computational resources but also yields inferior recovery performance. Although k=4 is slightly faster than k=8, we ultimately selected k=8 as the optimal k-nearest neighbors hyperparameter by considering both efficiency and accuracy.

5.3 CATH4.2 Experiment for Different Seeds

we have performed experiments with three different random seeds. To further quantify the sensitivity, we report the mean and standard deviation of recovery rates across these seeds. The results are as follows:

Table 7: Results Across Different Random Seeds

Seed	Recovery ↑			Perlexity ↓		
	All	Single	Short	All	Single	Short
2333	61.29	53.91	55.27	2.9387	3.3597	3.2639
927	62.39	54.53	55.29	2.9466	3.3091	3.2213
42	62.47	54.70	57.98	2.9705	3.2939	3.1225

Based on the results above, we have updated the data in the model comparison to reflect the mean ± standard deviation across multiple random seeds. The following is a comparison table of different models. To maintain consistency with the previous approach in the main text, we only show the mean values in the table without displaying the standard deviations.

Table 8: CATH4.2 dataset experiments

Model	Perplexity ↓			Recovery ↑		
	Short	Single	All	Short	Single	All
GVP	7.23	7.84	5.36	30.60	28.95	39.47
GCA	7.09	7.49	6.05	32.62	31.10	37.64
StructGNN	8.29	8.74	6.40	29.44	28.26	35.91
ProteinMPNN	6.21	6.68	4.61	36.35	34.43	45.96
LM-design	6.77	6.46	4.52	37.88	42.47	55.65
ESM-IF	8.18	6.33	6.44	31.3	38.5	38.3
PiFold	6.04	6.31	4.55	39.84	38.53	51.66
SurfPro	–	–	3.13	–	–	57.78
Knowledge-Design	5.48	5.1	3.46	44.66	45.45	60.77
ScFold	5.80	5.99	4.61	41.60	40.10	52.22
SurfFold(our)	**3.20±0.06**	**3.32±0.03**	**2.95±0.01**	**56.18±1.27**	**54.38±0.34**	**62.05±0.54**

5.4 Ablation Experiment for Different Seeds

we have conducted three independent runs with different random seeds to assess the robustness of the results. The updated results, including the mean and standard deviation of performance metrics, are reported in the table below. In the main text of the paper, in order

Table 9: Ablation experiments

		SurfFold	Model1	Model2	Model3	Model4	Model5
Protein features	backbone	✓	✓	✓	✓	✓	✓
	side-chain	✓	✓			✓	✓
	surface	✓			✓	✓	✓
alignment Module	8 nearest alignment	✓			✓	✓	
	cross attention	✓			✓		✓
Results	Perplexity	2.95±0.01	3.23±0.02	13.27±0.36	8.20±0.06	3.24±0.02	3.12±0.03
	Recovery	62.05±0.54	56.94±0.62	18.31±1.52	36.03±0.96	57.61±0.53	60.04±1.21

to maintain consistency with previous works, we only display the average values of the results

in the table and do not show the standard deviation. Therefore, the results presented in the paper are based on a single run. In the updated results, we have now included the standard deviations to better showcase the stability of the model performance and the contribution of each component.

These results show that the performance difference between SurfFold (62.05% recovery) and Model5 (60.04% recovery) is statistically significant, considering the standard deviations. The ablation study confirms that the inclusion of the cross-attention mechanism and surface features contributes significantly to the improved performance, particularly in terms of recovery rate. The minor difference in recovery between Model5 and the full model in the initial single-run results can now be attributed to the actual contribution of the modules, rather than training fluctuations.

5.5 Case Study

We have Design case study for our methond. Specifically, we randomly selected three proteins from the test set and used both our SurfFold and PiFold models to predict their sequences. We then extracted fragments from the predictions. Additionally, we visualized the protein surface point clouds generated by the models and highlighted the surface points that had the greatest impact on the predictions. The results are shown in the Figure 2.

The experimental results indicate that the protein surface point cloud indeed plays a role in sequence prediction and contributes significantly to the accuracy of the prediction. However, based on the experiment, we found that not all surface point clouds were involved in the prediction. The model automatically selects the most relevant surface point clouds based on surface variations to predict the protein sequence.

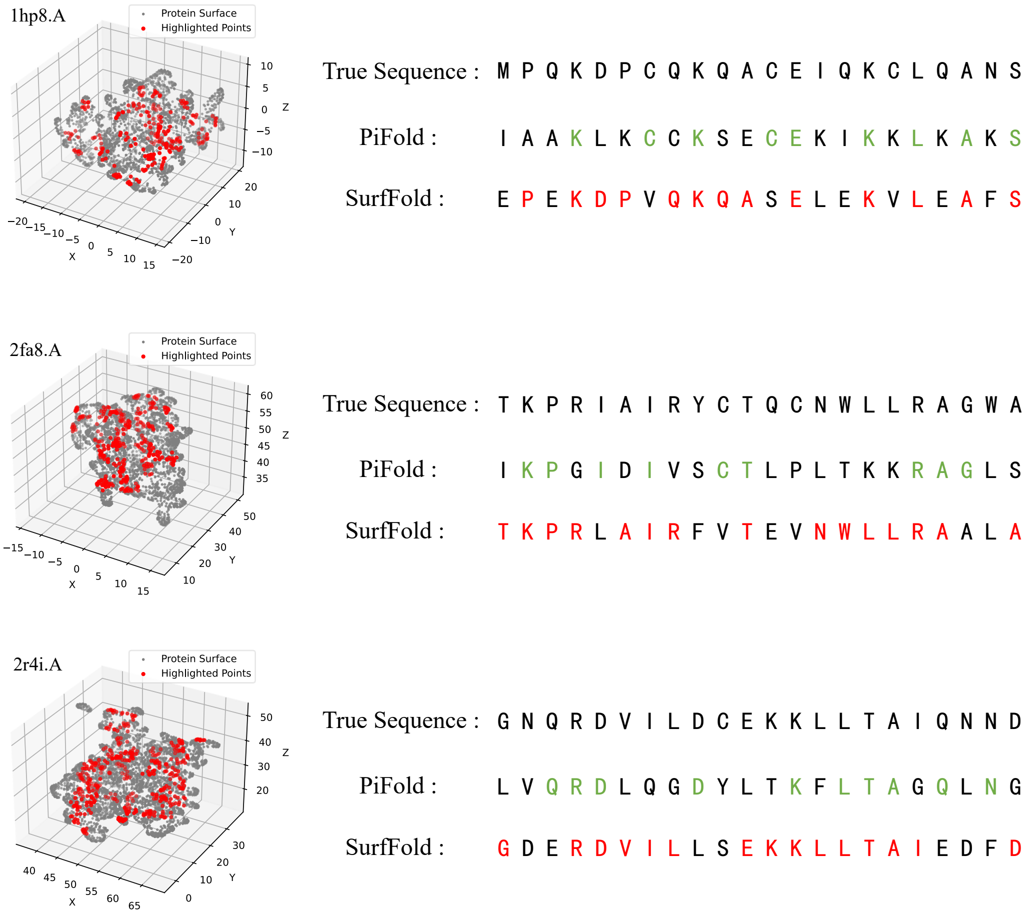

Figure 2: Case Study
